# Progress in Cancer

**Published:** 1903-04-18

**Authors:** 


### Progress in Cancer.

(*Concluded from page 26.*)

Wild [4] protests against the routine use of thyroid extract in a disease attended by progressive emaciation and exhaustion. It may hasten the end considerably without affecting the disease. According to Bland Sutton [5] secondary deposits are far more likely to occur in one or both ovaries than in the remaining breast after removal of a breast for cancer; and Allchin, commenting on this statement, considers the origin in the second breast to be primary rather than secondary, and advises immediate removal of it as in primary cancers. Observations upon the action of cytolytic serum in carcinoma are given by Von Leyden and Blumenthal.[6] They were unable to transfer human carcinoma to dogs, but in a few cases they were able to transfer canine carcinoma from one dog to another. They made an emulsion of growths extirpated from dogs and injected rabbits with it. The serum obtained from the rabbit they injected into another dog suffering from cancer as verified by microscope. Shrinking and softening of the growth resulted, and there was fatty degeneration of the cells with leucocytic infiltration, and subsequent disappearance. Another experiment in which the serum was obtained by compressing a removed tumour, was successful in reducing the growth in another dog. Three cases of human cancer were similarly treated. Growths freshly extirpated were compressed, and the serum injected into animals first to prove its harmlessness, and then into the patients. In the first and second cases, which were advanced and eventually terminated fatally, no metastases were observed, and glands inflamed and probably infiltrated disappeared. The third case was that of a woman aged 42, who was bedridden with extreme weakness resulting from cancer of the uterus. Serum obtained from a goat which had been previously injected with the cancer serum, was first used, and then the latter direct. The growth did not advance after this, and appeared to degenerate in parts. The patient was able to leave her bed for hours at a time at the end of 10 months' treatment. The authors are satisfied that these experiments are worth continuing.

Hoyten [7] has also injected the expressed juice of extirpated carcinomata into two cancerous patients. In both cases pain was relieved and the condition ameliorated, but both patients died subsequently. For pain in cancer Wild [8] speaks highly of phenacetin in the early cases. Later, when very severe, opium and morphia may be pushed to very large quantities. The best substitute for this when the dose is getting too great is 1-100th to 1-15th grain of hyoscine hydrobromide repeated as required for a few days, when the morphia can be again administered in smaller doses. He is satisfied that alcohol in carefully regulated doses is a most valuable drug in maintaining nutrition. Bryant [9] testifies to the application of *x*-rays in cancer relieving pain, and though local manifestations have disappeared under treatment, he would not regard this method of treatment as curative. He mentions several cases which have been ameliorated by these means, including two cases of cancerous stricture of the rectum, where not only pain was relieved, but the stricture relaxed sufficiently to allow of the painless passage of fæces. In these cases the rays were applied through the perineum. Dawson Turner [10] has obtained in 15 out of 18 cases treated by phototherapy diminution of pain, loosening of adhesions, and removal of cancerous and sarcomatous tumours. The cases treated were nearly all inoperable, and comprised 9 tumours of the breast which were the most successful, 2 of the tongue which were the least so, and one each in the scalp, penis with scrotum, cervix, rectum, larynx, glands in groin, and parotid gland. He looks upon the treatment as a race with the disease. Recurrences occur, but these yield to treatment, and in his cases a stage has been arrived at which one or two exposures a week have been sufficient to keep the disease in abeyance and to remove distressing symptoms. Brook [11] speaks of three cases of cancer of the breast in which *x*-rays were of service. The first was a recurrence in the scar after the breast had been removed. The nodules disappeared and the general condition improved greatly before death. In the other two all evidence of the local disease disappeared and the patients are apparently free of cancer.

Sarcomata [12] of the femur have a very fatal prognosis. Only 1 out of 15 cases in Coley's experience was yet alive at the time of reporting, and this involved the lower end of the bone for which amputation was performed at the hip. Of the tibia 5 out of 6 were dead. One which was treated with erysipelas toxins recovered. In the time of Gross the statistics showed more cures, possibly on account of sepsis. Wyeth suggests producing streptococcic infection in these cases without waiting for recurrence. Coley thinks an exploratory incision liable to set up a generalised infection. He prepares for the hip-joint amputation and after the application of the Esmarck, he then incises and makes a microscopic frozen preparation if necessary. McCosh has seen several cases where operation had failed to remove all the sarcomatous tissue and the patient recovered without recurrence.

Adami has classified tumours from an embryological standpoint; thus he speaks of tumours arising from the lining membranes of the body as lepidomata, and those from the mesodermic "pulp" tissue as hylomata. After the embryonic period the hylic tissues never take on the lepidic characters; the reverse of this is true for mature epithelium or hypothelium, but in the mesodermic substance there is less stability and tumours are apt to occur which Adami has termed transitional lepidomata. Starting from these classifications, Woolley [13] has examined specimens of each class to find if any difference exists between them in the arrangement of the finer supporting tissues. In carcinomata, using Mallory's method of staining, he has found as White found a stroma of white fibrous tissue outlining the cell-spaces, but with no intercellular network. He points out, however, that in rapidly growing and infiltrating cancers single cells may get isolated towards the margin of the growth giving a false appearance of an intercellular reticulum.

In Sarcomata, if gradually growing, fine fibrils of connective tissue may be seen between the cells, and this agrees with the findings of White and Johnston; but the latter states that such connective tissue fibrils between the cells is absent in endothelioma. Now, embryologically these latter tumours are very closely connected with sarcomata, more so than to carcinomata, but morphologically they more closely resemble carcinomata. Woolley finds on careful staining with Mallory's method, slightly altered in one particular, that these fibrils are to be found in endotheliomata, or rather a tendency to their formation, thus establishing the relation between these tumours and the sarcomata. His conclusion is that in the alveolar endotheliomata there is a partially formed intercellular reticular network, and that the infiltrating forms show a complete network which corresponds to that of the sarcomata; that all endotheliomata of whatever origin show a tendency to a sarcomatous structure as regards between cells and the reticulum. This may have some practical bearing upon the diagnosis of the more obscure growths.

[4] Brit. Med. Jour., Oct. 25. [5] Lancet, Oct. 18. [6] Brit. Med. Jour., Oct. 18. [7] Brit. Med. Jour., Oct. 25. [8] Brit. Med. Jour., Oct. 25. [9] Brit. Med. Jour., Oct. 25. [10] Brit. Med. Jour., Nov. 15. [11] Brit. Med. Jour., Oct. 25 [12] Annals of Surg., October, 1902. [13] Johns Hopkins Hosp. Bulletin, January, 1903.

